# Human Epidermal Growth Factor Receptor 2–Targeting [^68^Ga]Ga-ABY-025 PET/CT Predicts Early Metabolic Response in Metastatic Breast Cancer

**DOI:** 10.2967/jnumed.122.265364

**Published:** 2023-09

**Authors:** Ali Alhuseinalkhudhur, Henrik Lindman, Per Liss, Tora Sundin, Fredrik Y. Frejd, Johan Hartman, Victor Iyer, Joachim Feldwisch, Mark Lubberink, Caroline Rönnlund, Vladimir Tolmachev, Irina Velikyan, Jens Sörensen

**Affiliations:** 1 Division of Nuclear Medicine and PET, Department of Surgical Sciences, Uppsala University, Uppsala, Sweden;; 2 Department of Immunology, Genetics, and Pathology, Uppsala University, Uppsala, Sweden;; 3 Division of Radiology, Department of Surgical Sciences, Uppsala University, Uppsala, Sweden;; 4 Clinical Research and Development Unit, Uppsala University Hospital, Uppsala, Sweden;; 5 Affibody AB, Solna, Sweden;; 6 Department of Oncology–Pathology, Karolinska Institute, Stockholm, Sweden;; 7 Department of Clinical Pathology and Cancer Diagnostics, Karolinska University Hospital, Stockholm, Sweden; and; 8 Department of Medical Physics, Uppsala University Hospital, Uppsala, Sweden

**Keywords:** [^68^Ga]Ga-ABY-025, breast cancer, HER2 positive, affibody molecules, PET/CT

## Abstract

Imaging using the human epidermal growth factor receptor 2 (HER2)–binding tracer ^68^Ga-labeled Z_HER2:2891_-Cys-MMA-DOTA ([^68^Ga]Ga-ABY-025) was shown to reflect HER2 status determined by immunohistochemistry and in situ hybridization in metastatic breast cancer (MBC). This single-center open-label phase II study investigated how [^68^Ga]Ga-ABY-025 uptake corresponds to biopsy results and early treatment response in both primary breast cancer (PBC) planned for neoadjuvant chemotherapy and MBC. **Methods:** Forty patients with known positive HER2 status were included: 19 with PBC and 21 with MBC (median, 3 previous treatments). [^68^Ga]Ga-ABY-025 PET/CT, [^18^F]F-FDG PET/CT, and core-needle biopsies from targeted lesions were performed at baseline. [^18^F]F-FDG PET/CT was repeated after 2 cycles of therapy to calculate the directional change in tumor lesion glycolysis (Δ-TLG). The largest lesions (up to 5) were evaluated in all 3 scans per patient. SUVs from [^68^Ga]Ga-ABY-025 PET/CT were compared with the biopsied HER2 status and Δ-TLG by receiver operating characteristic analyses. **Results:** Trial biopsies were HER2-positive in 31 patients, HER2-negative in 6 patients, and borderline HER2-positive in 3 patients. The [^68^Ga]Ga-ABY-025 PET/CT cutoff SUV_max_ of 6.0 predicted a Δ-TLG lower than −25% with 86% sensitivity and 67% specificity in soft-tissue lesions (area under the curve, 0.74 [95% CI, 0.67–0.82]; *P* = 0.01). Compared with the HER2 status, this cutoff resulted in clinically relevant discordant findings in 12 of 40 patients. Metabolic response (Δ-TLG) was more pronounced in PBC (−71% [95% CI, −58% to −83%]; *P* < 0.0001) than in MBC (−27% [95% CI, −16% to −38%]; *P* < 0.0001), but [^68^Ga]Ga-ABY-025 SUV_max_ was similar in both with a mean SUV_max_ of 9.8 (95% CI, 6.3–13.3) and 13.9 (95% CI, 10.5–17.2), respectively (*P* = 0.10). In multivariate analysis, global Δ-TLG was positively associated with the number of previous treatments (*P* = 0.0004) and negatively associated with [^68^Ga]Ga-ABY-025 PET/CT SUV_max_ (*P* = 0.018) but not with HER2 status (*P* = 0.09). **Conclusion:** [^68^Ga]Ga-ABY-025 PET/CT predicted early metabolic response to HER2-targeted therapy in HER2-positive breast cancer. Metabolic response was attenuated in recurrent disease. [^68^Ga]Ga-ABY-025 PET/CT appears to provide an estimate of the HER2 expression required to induce tumor metabolic remission by targeted therapies and might be useful as an adjunct diagnostic tool.

## Visual Abstract

**Figure:**
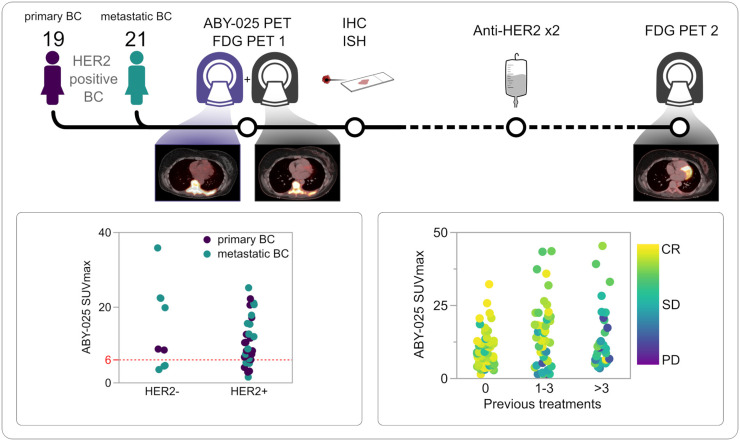

Up to 20% of breast cancer cases have human epidermal growth factor receptor 2 (HER2) overexpression with or without HER2 oncogene amplification (1). Treatment with HER2-targeted monoclonal antibodies, such as trastuzumab and pertuzumab (double blockage), in combination with chemotherapy is the standard of care in HER2-positive subtypes both in the neoadjuvant setting and in the recurrent or metastatic setting (2). HER2-targeted therapies act on the receptor level, and immunohistochemistry staining of a biopsied specimen is required to confirm sufficiently high HER2 expression in the neoadjuvant setting and is recommended in the metastatic setting, with or without in situ hybridization (ISH) (3). Even so, breast cancer is a heterogeneous disease, and intertumoral HER2 expression may vary within the same patient and over time (4).

Currently, treatment failure is relatively common, and cure remains rare in metastatic disease (5). Hence, the possibility to evaluate the HER2 status for the whole body is appealing (6). Efforts have been directed toward using radiolabeled scaffold proteins, such as affibody molecules, in combination with PET (7). An analog selected from a billion-entry library, Z_HER2:2891_-Cys-MMA-DOTA (ABY-025), demonstrated high affinity toward HER2, almost irreversible binding, and favorable pharmacokinetics in both preclinical and clinical settings (8–11).

ABY-025 was labeled with positron-emitting ^68^Ga (half-life, 67.6 min), allowing patient imaging for up to 4 h after injection (9*,*^10^*,*^12^). [^68^Ga]Ga-ABY-025 PET/CT showed strong potential in evaluating HER2 expression in patients with metastatic breast cancer (MBC) in an early-phase clinical study (10). In addition to establishing its safety and dose efficacy, the study found that uptake in tumor lesions correlated well with the biopsy-determined HER2 status. Cutoffs using an SUV_max_ of 6.0 and 8.0 at 2 and 4 h after injection, respectively, were suggested for stratifying HER2-positive lesions (10).

Trastuzumab resistance has been documented both as primary and as acquired, prominently in previously treated patients (13*,*^14^). With multiple lines of treatment currently available, early treatment response evaluation using [^18^F]F-FDG PET/CT has become a valuable tool to guide treatment regimens both in primary breast cancer (PBC) (15*,*^16^) and in MBC (17*,*^18^). This supports its use for early metabolic response evaluation.

The primary endpoint was to correlate [^68^Ga]Ga-ABY-025 uptake with biopsy immunohistochemistry staining in the therapy-naïve setting and in the recurrent metastatic setting. The key secondary endpoints were to investigate whether [^68^Ga]Ga-ABY-025 PET/CT predicts the treatment response and to investigate how previous treatments affect the outcome.

## MATERIALS AND METHODS

### Patient Population

The current study was a planned interim analysis of a phase II study embedded within a larger academically driven prospective open-label phase II and phase III diagnostic trial, approved by the Swedish Medical Products Agency (EudraCT 2017-002115-34; diarienummer, 5.1-2018-30296; ClinicalTrials.gov, NC-T03655353) and the Ethical Committee of Uppsala/Örebro county (2017-467). All patients gave written informed consent. Phase II of this study was initiated in September 2018 and included a total of 40 patients. The final study examination occurred in July 2021.

Women with newly diagnosed stage II or stage III PBC and planned for neoadjuvant therapy or women with confirmed progression in MBC and planned for HER2-targeted therapy concomitant with chemotherapy were considered as candidates for this study. The inclusion criteria included women with biopsy-confirmed HER2-positive or borderline HER2-positive breast cancer, at least 1 tumor lesion of at least 1.0 cm, at least 1 tumor available for biopsy, a negative pregnancy test and active contraceptive measures for women of child-bearing age, at least 18 y of age, a predicted survival of more than 12 wk, and a World Health Organization performance status of 2 or lower.

The exclusion criteria included women with biopsy-confirmed HER2-negative breast cancer before enrollment in this study; other coexisting malignancies; uncontrolled serious concomitant disease including congestive heart failure, inadequate organ function such as neutropenia, or abnormally high liver or kidney function tests (absolute neutrophil count < 1,500 cells/mm^3^; total bilirubin ≥ 1.5 times the upper limit of normal [unless the patient had Gilbert syndrome]; aspartate transaminase [serum glutamic oxaloacetic transaminase] or alanine transaminase [serum glutamic pyruvic transaminase] ≥ 5.0 times the upper limit of normal; serum creatinine clearance < 30 mL/min); those who were sexually active of child-bearing age and not willing to take proper contraceptive measures; and patients assessed by investigators to be unable or unwilling to comply with the protocol requirements.

Patients fulfilling the above-mentioned criteria underwent [^68^Ga]Ga-ABY-025 PET/CT, [^18^F]F-FDG PET/CT, and image-guided biopsies at baseline, followed by follow-up [^18^F]F-FDG PET/CT after 2 cycles of guideline-based HER2-targeted treatment.

### Production of [^68^Ga]Ga-ABY-025

The ABY-025 peptide was provided by Affibody AB. Production of [^68^Ga]Ga-ABY-025 was done by a fully automated labeling procedure using a disposable dedicated cassette system (Modular-Lab PharmTracer, Eckert & Ziegler GmBh) with a radiochemical purity of 98% ± 1%, as described previously (12).

### [^68^Ga]Ga-ABY-025 PET/CT

All scans were performed using a Discovery MI PET/CT scanner (GE Healthcare), and scans included a section from the skull apex to mid thigh. [^68^Ga]Ga-ABY-025 was injected intravenously (139 ± 43 MBq; 327 ± 29 μg of peptide), followed by PET/CT imaging at 3 h. Acquisition time was 4 min per bed position.

The first 10 patients were closely monitored by electrocardiograms and clinical examinations during their stay at the PET center. All patients received a phone call after 24 h from the time of [^68^Ga]Ga-ABY-025 administration to record any adverse effects.

### [^18^F]F-FDG PET/CT

[^18^F]F-FDG PET/CT was performed at baseline within 1 wk of the [^68^Ga]Ga-ABY-025 PET/CT scan (median, 1 d). A follow-up [^18^F]F-FDG PET/CT scan was performed after the second treatment cycle to assess treatment response. Contrast-enhanced diagnostic CT was performed as part of each [^18^F]F-FDG PET/CT protocol. Scans were conducted according to a standard clinical protocol with injection of 3 MBq of [^18^F]F-FDG per kilogram of body weight 1 h before the scan.

### PET Measurements

Images obtained from both [^68^Ga]Ga-ABY-025 PET/CT and [^18^F]F-FDG PET/CT were analyzed at Uppsala University Hospital using HYBRID 3D (HERMES Medical Solutions). SUVs were gathered for subsequent analysis. To assess the metabolic response, [^18^F]F-FDG PET/CT total lesion glycolysis (TLG; SUV_mean_ × metabolic tumor volume) was measured in up to 5 of the largest lesions per patient both at baseline and after 2 cycles of HER2-targeted treatment. The percentage change in TLG (Δ-TLG) was calculated as 100 × (TLG2 – TLG1)/TLG1. A Δ-TLG lower than −25% was considered to be a positive metabolic response to treatment. Clinical response was evaluated by the attending physician as part of the routine patient follow-up visits.

### Biopsies

Lesions were selected for study biopsies on the basis of uptake on both PET scans and accessibility at a multidisciplinary trial conference the day after the last baseline PET scan. The biopsied lesions were carefully located and manually defined on [^18^F]F-FDG PET/CT both at baseline and at follow-up using [^68^Ga]Ga-ABY-025 PET/CT as a reference. Biopsies taken (1 per patient; *n* = 40) were centrally analyzed (Karolinska University Hospital) using immunohistochemistry and ISH techniques. A biopsy sample was considered to be HER2-positive if the immunohistochemistry score was 3+ in more than 10% of the cell areas or 2+ in more than 10% of the cell areas with either a HER2/chromosome enumeration probe 17 ratio of at least 2.0 or a HER2 copy number of at least 6.0 by ISH. Biopsy results were considered to be borderline HER2-positive if the immunohistochemistry score was 2+, the HER2/chromosome enumeration probe 17 ratio was less than 2.0, or the HER2 copy number was 4.0–6.0 by ISH. Biopsy samples not fulfilling the above-mentioned criteria were considered to be HER2-negative. The term *HER2 status* was used to reflect either positive (HER2-positive) or negative (HER2-borderline/negative) expression.

### HER2-Targeted Treatment

Patients planned for neoadjuvant treatment and patients with first-time recurrent disease received trastuzumab, pertuzumab, and chemotherapy according to guidelines, whereas patients with multiple recurrent disease received trastuzumab emtansine.

### Statistical Analysis

PET metrics were reported as mean ± SD unless otherwise stated. Receiver operating characteristic curve analysis was used to investigate the predictive value of the variables and to define a cutoff for [^68^Ga]Ga-ABY-025 SUV_max_ determining HER2 positivity. Bivariate and multivariate analyses were used to investigate associations between PET metrics, biopsy results, and outcomes. Nonparametric ANOVA was used to investigate the means in patient groups stratified on the basis of the number of previous treatments. Statistical analyses were performed with Prism 8 (GraphPad Software) and JMP statistical software. A *P* value less than 0.05 was considered to be statistically significant.

## RESULTS

### Patient Characteristics

**FIGURE 1.**
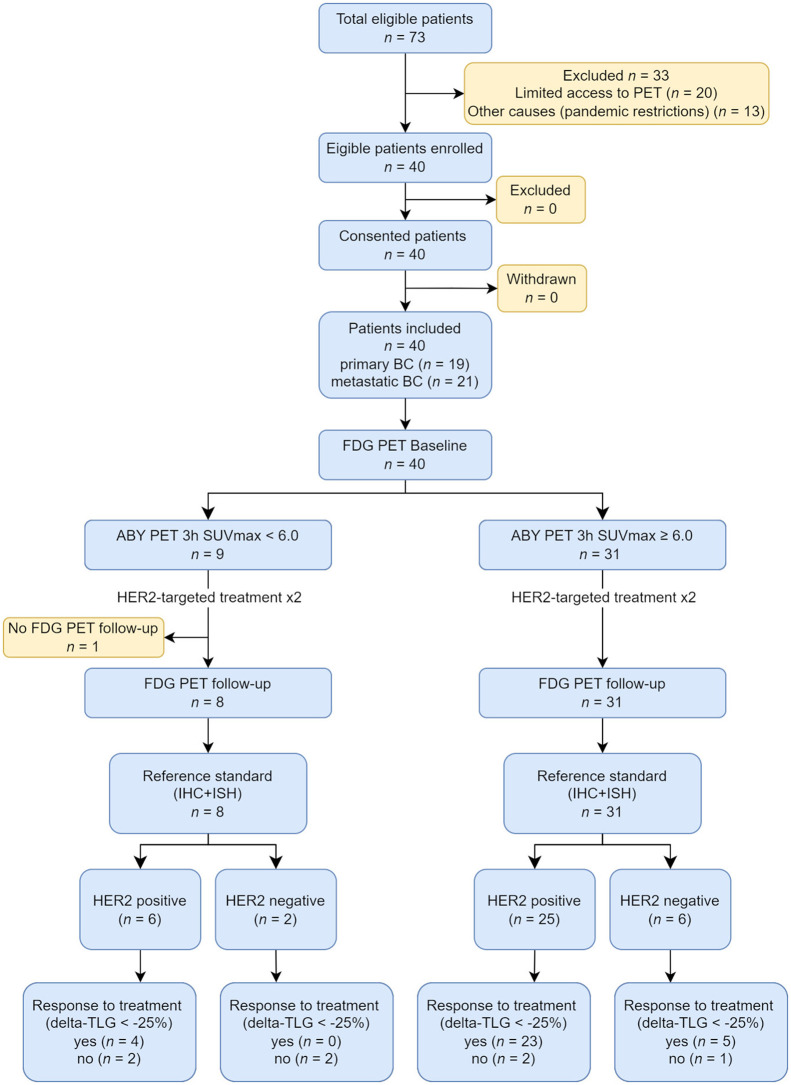
Diagram of recruitment and diagnostic classification of study subjects, according to the Standards for Reporting Diagnostic Accuracy. BC = breast cancer; ABY = [^68^Ga]Ga-ABY-025; IHC = immunohistochemistry.

**FIGURE 2.**
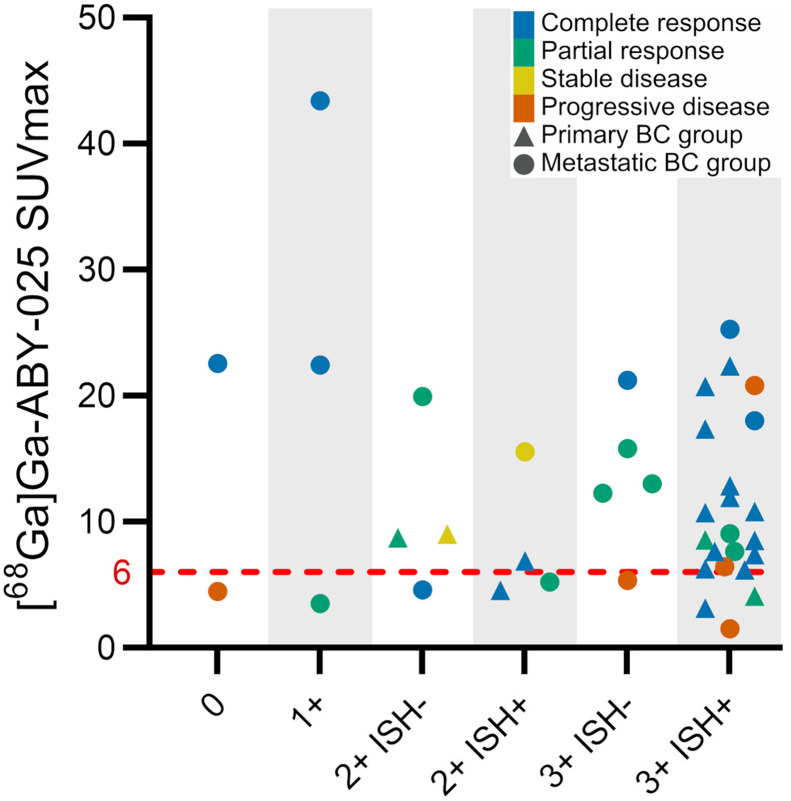
Color-coded clinical response in patients with breast cancer. *x*-axis is HER2 status according to immunohistochemistry with ISH from trial biopsies (*n* = 40). *y*-axis is corresponding uptake in biopsied lesions using [^68^Ga]Ga-ABY-025 PET/CT. Red line indicates SUV_max_ of 6.0. BC = breast cancer.

Patient characteristics and descriptive data are shown in Table 1. Forty patients were consecutively enrolled from September 2018 through July 2021. Nineteen patients had PBC, and 21 patients had MBC. Twenty patients were treatment-naïve; 12 had received 1–3 treatments, and 8 patients had received more than 3 treatments before this study. Biopsy results were HER2-positive in 31 patients, borderline HER2-positive in 3 patients, and HER2-negative in 6 patients (Table 2). All patients received HER2-targeted therapy during the trial with or without chemotherapy, except for 1 patient in the HER2-negative group, who received chemotherapy only. HER2-targeted therapy was given on the basis of the patients’ initial HER2 status before enrollment. The study flow diagram according to the Standards for Reporting Diagnostic Accuracy is shown in Figure 1. All patients completed all planned PET/CT scans. One lesion per patient was successfully biopsied and analyzed (Fig. 2). The tissue types of biopsied lesions and their corresponding HER2 status according to immunohistochemistry and ISH are summarized in Supplemental Table 1 (supplemental materials are available at http://jnm.snmjournals.org). One patient with MBC was diagnosed with breast cancer gene–mutated triple-negative disease from the trial biopsy (HER2 score, 2+; ISH, negative), after which neoadjuvant treatment and the subsequent [^18^F]F-FDG PET/CT were aborted and surgery performed instead. This patient had only faint [^68^Ga]Ga-ABY-025 uptake (SUV_max_, 4.6) in the tumor. There were no adverse events attributable to the study drug throughout the study.

**TABLE 1.** Patient Characteristics and Descriptive Data

	HER2 status*		
Parameter	Positive cases (*n* = 31)	Negative cases (*n* = 6)	Borderline cases (*n* = 3)
Median age (y)	57 (29–89)	63 (45–78)	58 (53–62)
Estrogen receptor–positive (≥10%)	14 (45%)	4 (67%)	2 (67%)
Stage			
II	14 (45%)	0	1 (33%)
III	2 (7%)	1 (17%)	0
IV	15 (48%)	5 (83%)	2 (67%)
Molecular subtype			
Luminal A		1 (17%)	
Luminal B		3 (50%)	2 (67%)
HER2-positive	17 (55%)		
HER2-positive/luminal	14 (45%)		
Triple-negative		2 (33%)	1 (33%)
Neoadjuvant treatment			
Primary	15 (48%)	1 (17%)	
Metastatic	2 (6%)		1 (33%)
Previous treatments			
PBC			
None	17 (55%)	1 (17%)	1 (33%)
MBC			
None			1 (33%)
1	1 (3%)	1 (17%)	0
2	5 (16%)	2 (33%)	0
3	3 (10%)	0	0
4	2 (6%)	1 (17%)	0
5	1 (3%)	1 (17%)	0
6+	2 (6%)	0	1 (33%)

* Based on immunohistochemistry and ISH results.

Qualitative data are number and percentage; continuous data are median and range.

**TABLE 2.** Anatomic Distribution and [^68^Ga]Ga-ABY-025 Mean SUV_max_ for Lesions Used in This Study

	HER2 status					
	Positive (*n* = 31)		Negative (*n* = 6)		Borderline (*n* = 3)	
Site of disease	Number	SUV_max_	Number	SUV_max_	Number	SUV_max_
Breast	26 (18)	10.1 ± 6.9	3 (1)	6.0 ± 2.4	2 (2)	6.8 ± 3.1
Axilla	22 (2)	10.9 ± 6.3	2	9.0 ± 3.7	—	
Liver	5 (3)	13.7 ± 6	6 (3)	18.1 ± 7	2 (1)	12.6 ± 10.5
Lung or distal lymph nodes	24 (5)	9.1 ± 7.4	3	5.3 ± 4.9	—	
Bone	16 (3)	16.2 ± 6.1	9 (1)	28 ± 14	4	11.9 ± 4.5
Other	8	15.8 ± 17	2 (1)	3.9 ± 0.4	—	

Number of biopsied lesions is in parentheses, 1 per patient. SUV_max_ is mean ± SD.

### Tumor Lesions

**FIGURE 3.**
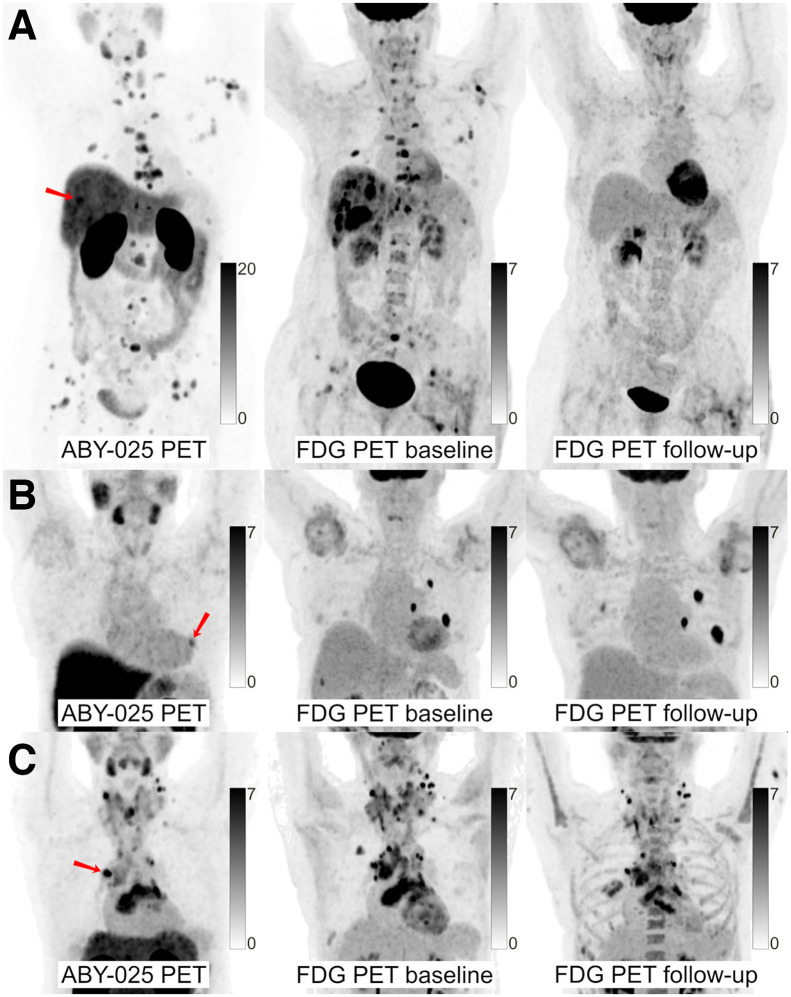
[^68^Ga]Ga-ABY-025 PET/CT and [^18^F]F-FDG PET/CT images at baseline with [^18^F]F-FDG PET/CT follow-up after 2 cycles of treatment in biopsy-confirmed HER2-positive disease. (A) Patient with high [^68^Ga]Ga-ABY-025 uptake (SUV_max_, 21), who previously received 3 lines of treatment. [^18^F]F-FDG PET/CT follow-up showed complete metabolic response. (B) Patient with low uptake (SUV_max_, 5.4), who previously received 3 lines of treatment. [^18^F]F-FDG PET/CT follow-up showed disease progression (Δ-TLG, +68%) despite HER2-targeted treatment. (C) Patient with heterogeneous [^68^Ga]Ga-ABY-025 uptake, who previously received 7 lines of treatment. [^18^F]F-FDG PET/CT follow-up showed heterogeneous response, with lesions higher in [^68^Ga]Ga-ABY-025 uptake tending to have better response. Arrows indicate biopsy sites.

We measured tracer uptake in up to 5 of the largest lesions per patient, including the biopsied lesion. Anatomic distribution of measured lesions is shown in Table 2. In total, 134 lesions were included in the analysis: 31 breast lesions, 27 lung lesions, 29 bone lesions, 24 axillary lymph nodes, 13 liver lesions, and 10 in other sites, such as muscle or remote lymph nodes. [^68^Ga]Ga-ABY-025 SUV_max_ variation at the intrapatient level was observed, with ranges among different lesions within 1 patient exceeding 10.0 in 11 patients (mean, 7.6; range, 1.2–39.1; Fig. 3C).

### [^68^Ga]Ga-ABY-025 Uptake Compared with HER2 Status

In total, 12 patients showed a mismatch between PET and HER2 status, using SUV_max_ of 6.0 as a cutoff to distinguish HER2-positive from HER2-negative expression (Fig. 2). No significant association between [^68^Ga]Ga-ABY-025 uptake and HER2 status was found (*P* = 0.13).

### Clinical and Metabolic Response

Thirty-two patients had either partial or complete metabolic response after receiving 2 cycles of treatment. Clinically, 22 patients had complete response, 11 had partial response, 2 had stable disease, and 5 had progressive disease. Global Δ-TLG was significantly associated with the clinical response (*P* < 0.0001).

**FIGURE 4.**
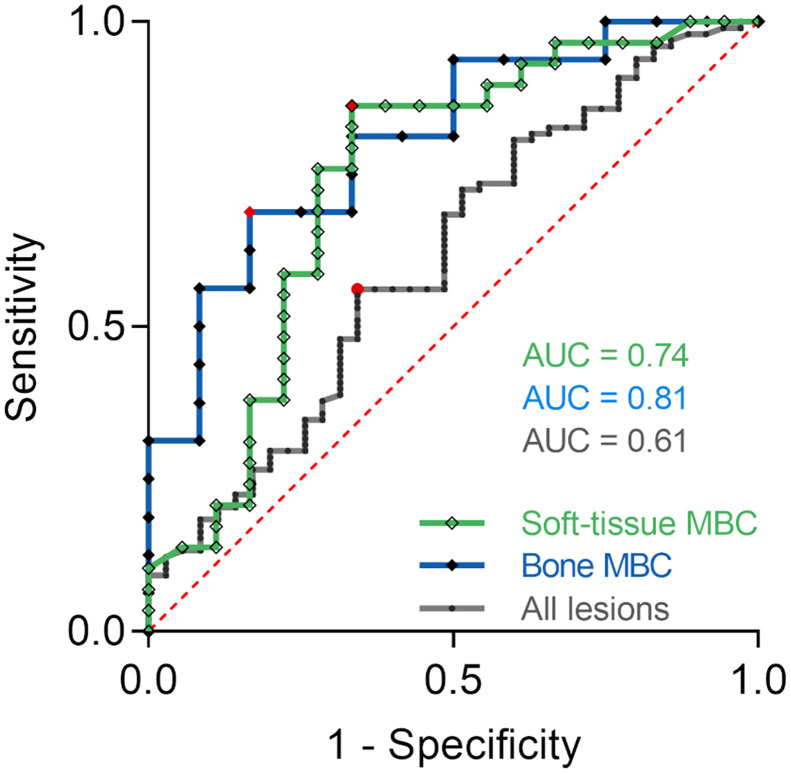
Receiver operating characteristic curve analysis of positive metabolic response after 2 cycles of HER2-targeted treatment in breast cancer patients according to [^68^Ga]Ga-ABY-025 uptake. Optimal SUV_max_ cutoff (red circle and diamonds) was 6.0 (*n* = 47; *P* = 0.01) in soft-tissue metastases (green line), 16.2 (*n* = 28; *P* = 0.003) in skeletal metastases (blue line), and 10.7 (*n* = 133, *P* = 0.03) in all lesions (black line). Sensitivity and specificity were 86% and 67%, 69% and 83%, and 56% and 66%, respectively. Red line represents receiver operating curve for random guess. At least 25% reduction in Δ-TLG was considered metabolic response. AUC = area under curve.

[^68^Ga]Ga-ABY-025 uptake predicted metabolic response after 2 cycles of treatment measured as Δ-TLG below −25%, with a best cutoff SUV_max_ of 10.7 in all patients (area under the curve [AUC], 0.61; 56% sensitivity; 66% specificity; *P* = 0.03; Fig. 4). Invasive HER2 status of all 40 biopsied lesions showed 79% sensitivity and 37% specificity (AUC, 0.58; *P* = 0.06) in all patients. This was a prespecified analysis, which was followed by post hoc analyses. In the MBC group, a cutoff SUV_max_ of 10.9 increased the accuracy (AUC, 0.72; 71% sensitivity; 67% specificity; *P* < 0.001), whereas invasive HER2 status had 71% sensitivity and 40% specificity (AUC, 0.56; *P* = 0.3) in MBC.

A previously proposed SUV_max_ cutoff of 6.0 in MBC soft-tissue lesions yielded 86% sensitivity and 67% specificity (AUC, 0.74 [95% CI, 0.67–0.82]; *P* = 0.01) for prediction of positive metabolic response (Fig. 4). In skeletal lesions, a best cutoff SUV_max_ of 16.2 was significantly higher than in soft-tissue lesions (AUC, 0.81 [95% CI, 0.74–0.87]; 69% sensitivity; 83% specificity; *P* = 0.003) (Fig. 4). Again, invasive HER2 status did not achieve a significant predictive value (AUC, 0.56; *P* = 0.5). Neither [^68^Ga]Ga-ABY-25 uptake nor invasive HER2 status had a predictive value in PBC (*P* = 0.3).

**FIGURE 5.**
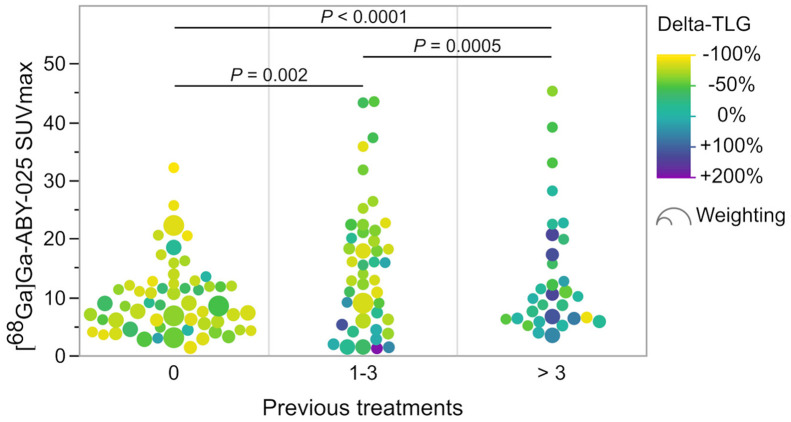
Number of previous treatments and their effect on response rate measured as Δ-TLG after 2 cycles of HER2-targeted treatment. One-way ANOVA showed significantly different response rates among 3 groups (*P* < 0.0001). Marker size reflects statistical weight of lesions per patient. *P* values represent Wilcoxon signed-rank test with regard to metabolic response among 3 groups.

A multivariate model for patient-level prediction of global Δ-TLG using the number of previous treatments, global TLG at baseline, [^68^Ga]Ga-ABY-025 SUV_max_, and invasive HER2 status in biopsied lesions as covariates showed independent significance for the number of previous treatments (*P* = 0.0004) and SUV_max_ in biopsied lesions (*P* = 0.018) but not for biopsy-derived HER2 status (*P* = 0.09) or baseline TLG (*P* = 0.17; *n* = 39). Δ-TLG on the lesional level remained significantly associated with [^68^Ga]Ga-ABY-025 uptake (*P* = 0.0009) when adjusted for the number of previous treatments, and this model achieved *R*^2^ of 0.30 (*n* = 133; Fig. 5). Global Δ-TLG per patient is listed in Supplemental Table 2.

In contrast to MBC, all PBC patients achieved metabolic response regardless of [^68^Ga]Ga-ABY-025 uptake; the average Δ-TLG was −27% (95% CI, −16% to −38%) in MBC and −71% (95% CI, −58% to −83%) in PBC (*P* < 0.0001). However, [^68^Ga]Ga-ABY-025 uptake was similar in both PBC and MBC, with a mean SUV_max_ of 9.8 (95% CI, 6.3–13.3) and 13.9 (95% CI, 10.5–17.2), respectively (*P* = 0.10).

Δ-TLG was significantly associated with the number of previous treatments. All therapy-naïve patients achieved a metabolic response regardless of the [^68^Ga]Ga-ABY-025 uptake. Patients with more than 3 previous treatments generally had poor metabolic responses, even when high HER2 availability by PET was present (Fig. 5). Patients with 1–3 previous treatments showed significantly better responses than patients with more than 3 previous treatments (*P* = 0.0005).

## DISCUSSION

The potential of [^68^Ga]Ga-ABY-025 PET/CT to quantify HER2 expression and to predict the treatment response was investigated in terms of associations of lesional [^68^Ga]Ga-ABY-025 uptake with HER2 status and metabolic response. To summarize, [^68^Ga]Ga-ABY-025 uptake correlated significantly with metabolic response on the patient level, particularly evident in the MBC group. However, there was no significant association of [^68^Ga]Ga-ABY-025 uptake with biopsy-derived HER2 status, and the latter was not significantly associated with a treatment response. Treatment response, on the other hand, was significantly associated with the number of previous treatments received. All treatment-naïve patients had statistically significant metabolic and clinical responses regardless of [^68^Ga]Ga-ABY-025 uptake.

The results showed that immunohistochemistry staining did not always reflect the biologic availability of the receptors; a HER2-positive biopsy sample with low [^68^Ga]Ga-ABY-025 uptake could be explained by obstacles with tracer binding (14), potentially also affecting the access of trastuzumab to the receptor, and a positive biopsy with negative PET was associated with poor outcome in the MBC group. A negative biopsy with high PET uptake is most likely explained by sampling errors or heterogeneous intratumoral receptor expression. A negative biopsy with positive PET was more pronounced in liver and skeletal lesions, which are technically more difficult to target by image-guided needle biopsies, and was not encountered in any of the biopsies from the PBC lesions. We recorded 4 examples of clinical complete response in MBC patients with a negative biopsy and positive PET (Supplemental Fig. 1), indicating biopsy sampling errors as a likely source of discrepancy. Similarly, other HER2-targeted PET tracers also showed a discrepancy in uptake compared with the biopsy samples (19).

We found an inverse association between the number of previous treatments and the metabolic response to current treatment. The more treatments previously received, the higher the [^68^Ga]Ga-ABY-025 uptake required to induce a metabolic response (Fig. 5). On a lesional level, [^68^Ga]Ga-ABY-025 uptake and the number of previous treatments in combination explained 30% (*R*^2^ = 0.30) of the metabolic response. The remaining 70% could be due to, among other factors, clonal variation of drug resistance to HER2-targeted therapy as a consequence of exposure to multiple previous treatments (13*,*^14^). It is worth mentioning that in-patient heterogeneity had a direct effect on evaluating the clinical treatment response, as some patients progressed despite HER2-targeted treatment, likely caused by clonal differentiation (Fig. 2). On the other hand, all therapy-naïve patients responded to treatment but to a variable extent and independently of PET-defined receptor availability, suggesting that primary resistance mechanisms were present in some untreated patients.

Predicting positive metabolic response to HER2-targeted therapy in lymph nodes and soft-tissue lesions was possible using a prespecified cutoff SUV_max_ of 6.0. However, in skeletal metastases, a higher cutoff value (SUV_max_, 16.2) was observed (Fig. 4). Skeletal metastases were generally found in MBC patients, and the high cutoff value could reflect an aspect of treatment resistance; however, Δ-TLG could also be confounded by a metabolic flare effect as a consequence of an inflammatory response in bone lesions (17). There were too few liver metastases in this cohort to evaluate a potential correlation of [^68^Ga]Ga-ABY-025 uptake with a reduction in metabolic response. No subject was diagnosed with brain metastasis.

The ability of [^68^Ga]Ga-ABY-025 PET/CT to provide a whole-body visualization of HER2 expression and to predict metabolic response is advantageous (Figs. 3A and 3B) and exceeded the biopsy-based approach in this cohort. The data available so far do not allow us to claim PET’s superiority, but broader access to HER2-targeted imaging will be needed if similar results are found in larger future trials. In particular, HER2-based imaging tools might provide a solution in situations where biopsies cannot be performed safely or when biopsy results are inconsistent. A PET-based approach to evaluate the appropriateness of targeted therapies in heterogeneous disease and early therapy evaluation might help avoid unnecessary side effects and might provide a more personalized opportunity for timely therapy corrections. The latter is becoming increasingly relevant, as multiple lines of treatments are currently available or in development (20*,*^21^). Biopsies remain indispensable to assess other targetable disease mechanisms and to identify patient-specific resistance mechanisms.

This study was initially intended as a multicentric phase II and phase III study, and the current data are the results from a prespecified interim analysis after inclusion of 40 patients. Unfortunately, the coronavirus disease 2019 pandemic coincided with the study, delaying our inclusion rate and prohibiting wider inclusion. In effect, intersite reproducibility remains to be studied. As we could not show a significant association between biopsy results and PET, which was the main endpoint, the planned phase III trial was aborted. Instead, further trials should focus on changes in clinical management and outcome using HER2 imaging.

## CONCLUSION

Immunohistochemistry staining and ISH are currently the gold standards to determine HER2 status in breast cancer. However, limitations in the metastatic setting still hinder accurate biopsy-based evaluation of heterogeneous HER2 expression. Hence, the advantage of noninvasive techniques such as [^68^Ga]Ga-ABY-025 PET/CT prevails. Although no correlation was found between biopsy results and [^68^Ga]Ga-ABY-025 PET/CT uptake, the latter showed a favorable predictive value in patients with MBC receiving HER2-targeted treatment. Patients exposed to multiple previous treatments were less likely to respond to treatment, and higher [^68^Ga]Ga-ABY-025 uptake was needed to induce a metabolic response.

## DISCLOSURE

This work was partially supported by grants from the Swedish Breast Cancer Association, Swedish Cancer Foundation (19 0507 Pj), Roche AB Sweden, and the Percy Falk Foundation. Fredrik Frejd and Joachim Feldwisch are employees and own shares in Affibody AB. Jens Sörensen received clinical advisor remunerations from Affibody AB. Johan Hartman obtained speaker’s honoraria or advisory board remunerations from Roche, Novartis, Pfizer, Eli Lilly, MSD, Veracyte, and ExactSciences and received institutional research support from Cepheid, Roche, and Novartis. No other potential conflict of interest relevant to this article was reported.

## Notes

KEY POINTS **QUESTION:** Does [^68^Ga]Ga-ABY-025 PET/CT show predictive value concerning treatment outcomes in patients with recurrent breast cancer? **PERTINENT FINDINGS:** [^68^Ga]Ga-ABY-025 PET/CT showed significant predictive value in the recurrent breast cancer group. **IMPLICATIONS FOR PATIENT CARE:** [^68^Ga]Ga-ABY-025 PET/CT enabled quantification of HER2 expression and prediction of treatment outcomes in patients with recurrent breast cancer.
